# *ABO* Genotype, ‘Blood-Type’ Diet and Cardiometabolic Risk Factors

**DOI:** 10.1371/journal.pone.0084749

**Published:** 2014-01-15

**Authors:** Jingzhou Wang, Bibiana García-Bailo, Daiva E. Nielsen, Ahmed El-Sohemy

**Affiliations:** Department of Nutritional Sciences, Faculty of Medicine, University of Toronto, Toronto, Ontario, Canada; The University of Manchester, United Kingdom

## Abstract

**Background:**

The ‘Blood-Type’ diet advises individuals to eat according to their ABO blood group to improve their health and decrease risk of chronic diseases such as cardiovascular disease. However, the association between blood type-based dietary patterns and health outcomes has not been examined. The objective of this study was to determine the association between ‘blood-type’ diets and biomarkers of cardiometabolic health and whether an individual's *ABO* genotype modifies any associations.

**Methods:**

Subjects (n = 1,455) were participants of the Toronto Nutrigenomics and Health study. Dietary intake was assessed using a one-month, 196-item food frequency questionnaire and a diet score was calculated to determine relative adherence to each of the four ‘Blood-Type’ diets. ABO blood group was determined by genotyping rs8176719 and rs8176746 in the *ABO* gene. ANCOVA, with age, sex, ethnicity, and energy intake as covariates, was used to compare cardiometabolic biomarkers across tertiles of each ‘Blood-Type’ diet score.

**Results:**

Adherence to the Type-A diet was associated with lower BMI, waist circumference, blood pressure, serum cholesterol, triglycerides, insulin, HOMA-IR and HOMA-Beta (P<0.05). Adherence to the Type-AB diet was also associated with lower levels of these biomarkers (P<0.05), except for BMI and waist circumference. Adherence to the Type-O diet was associated with lower triglycerides (P<0.0001). Matching the ‘Blood-Type’ diets with the corresponding blood group did not change the effect size of any of these associations. No significant association was found for the Type-B diet.

**Conclusions:**

Adherence to certain ‘Blood-Type’ diets is associated with favorable effects on some cardiometabolic risk factors, but these associations were independent of an individual's ABO genotype, so the findings do not support the ‘Blood-Type’ diet hypothesis.

## Introduction

A link between ABO blood group and diet was proposed by P.J. D'Adamo in his book “*Eat Right For Your Type*” published in 1996 [1]. The ‘Blood-Type’ diets have gained widespread attention from the public with more than 7 million copies sold in over 60 languages, and making the *New York Times* bestseller list [2]. D'Adamo postulates that the ABO blood group reveals the dietary habits of our ancestors and adherence to a diet specific to one's blood group can improve health and decrease risk of chronic diseases such as cardiovascular disease. Based on the ‘Blood-Type’ diet theory, group O is considered the ancestral blood group in humans so their optimal diet should resemble the high animal protein diets typical of the hunter-gatherer era. In contrast, those with group A should thrive on a vegetarian diet as this blood group was believed to have evolved when humans settled down into agrarian societies. Following the same rationale, individuals with blood group B are considered to benefit from consumption of dairy products because this blood group was believed to originate in nomadic tribes. Finally, individuals with an AB blood group are believed to benefit from a diet that is intermediate to those proposed for group A and group B [1]. The ‘Blood-Type’ diet also proposes that lectins, which are sugar-binding proteins found in certain foods [3], could cause agglutination if they are not compatible with an individual's ABO blood group.

The ABO blood group is a classification of blood based on the structural variation of a certain carbohydrate antigenic substance on red blood cells. As one of the first recognizable genetic variants in humans, the ABO blood group has been studied extensively for its association with a variety of diseases including cancer [4], [5], [6], [7], malaria [8], and cholera [9]. Regarding cardiometabolic diseases, individuals with blood group O were found to have lower levels of von Willebrand factor (VWF) [10] and had a reduced risk of venous thromboembolism compared to the other blood groups [11]. Furthermore, group B individuals were found to have lower levels of E-selectin [12] and a lower risk of type 2 diabetes compared to group O [13]. These findings demonstrate the potential importance of the ABO blood group in altering risk of disease, including cardiometabolic disease. However, little is known about whether the ABO blood group modifies an individual's response to diet. A recent systematic review concluded that no evidence exists to support the proposed health benefits of ‘Blood-Type’ diets [14]. Considering the lack of scientific evidence and the popularity of the ‘Blood-Type’ diet, the objective of this study was to determine the association between ‘Blood-Type’ diets and biomarkers of cardiometabolic health and whether an individual's *ABO* genotype modifies any associations.

## Materials and Methods

### Ethics statement

The study protocol was approved by the Research Ethics Board at the University of Toronto, and all subjects provided written informed consent.

### Participants

Subjects (n = 1,639) were participants of the Toronto Nutrigenomics and Health (TNH) Study, which is a cross-sectional examination of young adults aged 20 to 29 years. All subjects were recruited between October 2004 and December 2010 and completed a general health and lifestyle questionnaire, which included information on age, sex, ethnocultural group and other subject characteristics. Subjects who were likely under-reporters (less than 800 kcal per day) or over-reporters (more than 3,500 kcal per day for females or 4,500 kilocalories per day for males) of energy intake were excluded from the analyses. Subjects were also excluded if they had missing data for any of the biomarkers of interest or *ABO* genotype (n = 184). After exclusions, 1,455 subjects (993 women and 462 men) remained. Individuals were categorized into four major ethnocultural groups: White (n = 703), East Asians (n = 491), South Asians (n = 155), and others (n = 106).

### Dietary adherence score assessment

Dietary intake was assessed by a one-month, Toronto-modified Willet 196-item semi-quantitative food frequency questionnaire (FFQ) as described previously [15]. Briefly, each subject was given instructions on how to complete the FFQ by using visual aids of portion sizes to improve the measurement of self-reported food intake. Subject responses to the individual foods were converted into daily number of servings for each item. In order to quantify the adherence to each of the four ‘Blood-Type’ diets, four different diet scores were given to each subject regardless of his or her own blood group. Based on the food items listed in the ‘Blood-Type’ diets [1], subjects received one positive point for consuming one serving of each recommended food item and one negative point for consuming one serving of an item on the list of foods to avoid. Foods that are listed as “Neutral” were not included in the equation and do not contribute to the final score. The lists of recommended foods to eat or avoid for each ABO blood group are shown in the **Appendix S1**. Subjects were then grouped into tertiles based on their scores for each diet, with the top tertile representing those whose diet most closely resembles the corresponding ‘Blood-Type’ diet.

### Cardiometabolic risk factor assessment

Anthropometric measurements including height, weight, blood pressure and waist circumference were determined as previously described [16]. Body mass index (BMI; kg/m^2^) was calculated and physical activity was measured by questionnaire and expressed as metabolic equivalent (MET)-hours per week, as described previously [16], [17]. Overnight 12-hour fasting blood samples were collected to measure serum biomarkers of cardiometabolic disease including triglycerides, free fatty acids, C-reactive protein, glucose, insulin, and total-, HDL- and LDL-cholesterol, as described previously [15]. The homeostasis model of insulin resistance (HOMA-IR) was calculated by using the formula: (insulin * glucose)/22.5, and the homeostasis model of beta-cell function (HOMA-Beta) was calculated by using the formula: (20 * insulin)/(glucose - 3.5).

### ABO genotype identification

The Sequenom MassArray® multiplex method was used to determine the blood group of study participants by genotyping two single nucleotide polymorphisms (SNPs) (rs8176719Del>G; rs8176746A>C) in the *ABO* gene. The rs8176719 SNP indicates O-allele-specific 261delG while rs8176746 determines the galactose specificity of the encoded A/B transferases and thus the expression of A and B antigens on erythrocytes [18].

### Statistical analyses

Statistical analyses were performed using the Statistical Analysis Systems (SAS) Software program (version 9.2; SAS Institute Inc., Cary, North Carolina). The *a* error was set at 0.05 and reported p-values are 2-sided. Variables that were not normally distributed were either log_e_ or square root transformed prior to analysis, but the mean values and standard errors are displayed without transformation to facilitate interpretation. Subject characteristics were compared across ABO blood groups by using chi-square tests for categorical variables and analysis of covariance (ANCOVA) for continuous variables. ANCOVA was also used to compare means of biomarkers of cardiometabolic disease risk across tertiles of diet scores. Means compared between groups were adjusted for multiple comparisons using the Tukey-Kramer procedure. Age, sex, ethnocultural group and energy intake were used as covariates in the ANCOVA analysis. Physical activity and smoking were also considered, but not included in the final model because they did not significantly (P<0.05) alter the results. The p-values for the associations between ‘Blood-Type’ diet and cardiometabolic biomarker profile remained significant (P<0.001) regardless of whether or not these two variables were included in the model. To determine whether matching the blood group with the corresponding diet was associated with a more favorable cardiometabolic disease risk profile, we stratified the entire population into two groups; one with the matched blood group for the diet, and the other unmatched. We next examined the interaction between diet score and the matching status on levels of each cardiometabolic disease risk factor for each ‘Blood-Type’ diet by using the Tukey-Kramer correction. When a significant interaction effect was observed, we further compared the differences in the outcome between subjects with the matched blood group and the unmatched group in each of the tertiles of diet score.

## Results

Subject characteristics based on the ABO blood group are summarized in **Table 1**. After adjusting for age, sex, and ethnocultural group, subject characteristics were similar across ABO blood groups, except for insulin, HOMA-IR and HOMA-Beta (p<0.05). Although the overall association between blood group and total cholesterol was significant (p = 0.043), no difference was observed among specific ABO blood group.

**Table 1:** Subject Characteristics by ABO Genotype^a^.

	Genotype				
Characteristic	O	A	B	AB	P-value
Subjects [n (% of total)]	543 (37)	544 (38)	277 (19)	91 (6)	
Age (y)	22.7±2.5^b^	22.8±2.5	22.4±2.3	22.5±2.6	0.13
Sex [n (%)]					0.31
Female	358 (36)	387 (39)	187 (19)	61 (6)	
Male	185 (40)	157 (34)	90 (19)	30 (7)	
Ethnocultural group [n (%)]					<0.001^c^
White	267 (38)	307 (44)	93 (13)	36 (5)	
East Asian	167 (34)	166 (34)	125 (25)	33 (7)	
South Asian	58 (37)	40 (26)	45 (29)	12 (8)	
Others	51 (48)	31 (29)	14 (13)	10 (10)	
Body mass index (kg/m^2^)	23.5±0.2	23.3±0.2	23.4±0.2	24.1±0.4	0.16
Systolic blood pressure (mm Hg)	116.9±0.5	116.8±0.5	116.1±0.6	116.9±1.0	0.72
Diastolic blood pressure (mm Hg)	70.7±0.4	69.7±0.4	70.0±0.5	70.4±0.8	0.25
Waist circumference (cm)	75.1±0.4	73.9±0.4	73.5±0.6	74.9±1.0	0.28
Glucose (mmol/L)	4.86±0.02	4.86±0.02	4.84±0.02	4.85±0.04	0.95
Insulin (pmol/L)	47.3±1.7	53.5±1.8	54.8±2.2	49.0±3.7	<0.001^d^
HOMA-IR	1.44±0.05	1.64±0.06	1.68±0.07	1.49±0.12	<0.001^d^
HOMA-Beta	101.1±3.5	114.6±3.8	116.4±4.7	105.7±7.8	<0.001^d^
Total cholesterol (mmol/L)	4.17±0.04	4.29±0.04	4.18±0.05	4.17±0.08	0.045^e^
HDL cholesterol (mmol/L)	1.44±0.02	1.48±0.02	1.46±0.02	1.45±0.04	0.16
LDL cholesterol (mmol/L)	2.28±0.03	2.37±0.03	2.28±0.04	2.30±0.07	0.06
Total/HDL cholesterol	2.93±0.03	2.86±0.03	2.9±0.05	2.89±0.08	0.91
Triglycerides (mmol/L)	0.98±0.02	0.96±0.02	0.97±0.03	0.93±0.05	0.53
hs-CRP (mg/L)	1.34±0.12	1.36±0.13	1.24±0.17	1.78±0.27	0.35
Free fatty acids (μmol/L)	466.4±12.1	467.6±12.8	459.3±16.1	465.9±26.5	0.94

^a^ HDL, high density lipoprotein; LDL, low density lipoprotein; hs-CRP, high-sensitivity C-reactive protein. HOMA-IR, homeostasis model of insulin resistance; and HOMA-Beta, homeostasis model of beta-cell function. Differences across blood groups were assessed using χ2 test for categorical variables and ANCOVA for continuous variables with adjustment for age, sex, ethnocultural group and energy intake.

^b^ Mean ± SE (all such values).

^c^ Overall comparison is significantly different after a Tukey-Kramer correction (P<0.05).

^d^ (Blood Group A, Group B) > Group O after a Tukey-Kramer correction (P<0.05).

^e^ Overall comparison is significantly different after a Tukey-Kramer correction (P<0.05).

**Figure 1:**
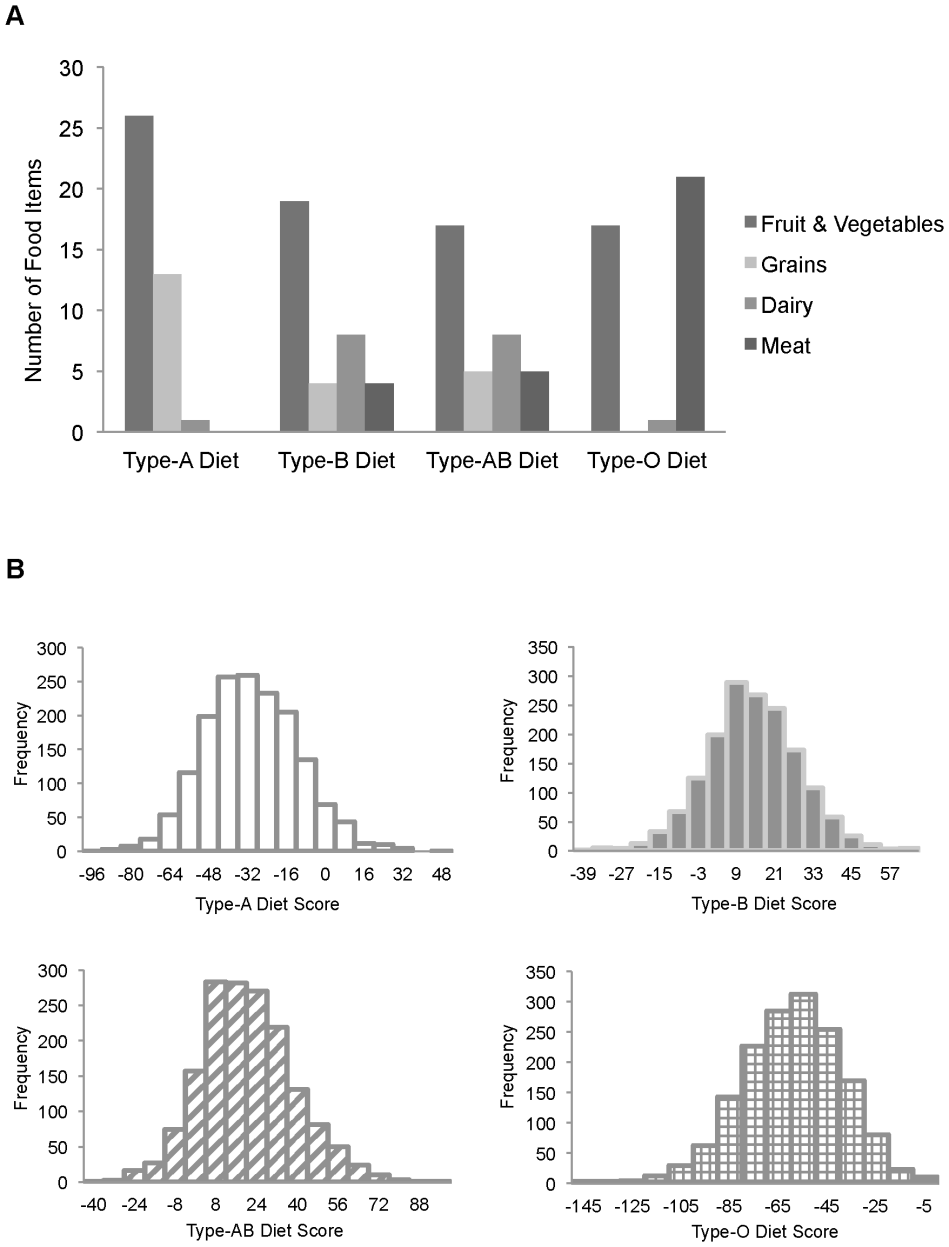
‘Blood-Type’ diet (A). Diet score distribution for each ‘Blood-Type’ diet (B).

Each ‘Blood-Type’ diet was first examined in the entire population without considering ABO blood groups. **Figure 1A** shows the total number of recommended items that were included in the FFQ for each diet. Briefly, the Type-A diet recommends high consumption of grains, fruits, and vegetables. The Type-B diet recommends high intakes of dairy products and moderate intakes of other food groups. The Type-AB diet is similar to the Type-B diet, but has more restrictions on specific food items. For example, only eggs and fish are recommended as sources of meat for group AB individuals (**Appendix S1**). The Type-O diet promotes high consumption of meats and avoidance of grain products. **Figure 1B** shows the diet score distribution. All four scores were normally distributed and did not require any transformation.

Characteristics of each ‘Blood-Type’ diet according to tertile of diet score are summarized in **Table S1.** Consistent with its recommendations, subjects in the highest tertile of the Type-A diet score consumed more fruits and vegetables and less meat (P<0.001). As for the two diets that recommend dairy consumption, high adherences to the Type-B and Type-AB diets were associated with higher intakes of dairy products (P<0.05). The dietary intake of those following the Type-O diet was also consistent with the diet's recommendations where more meat and less grain products were consumed as individuals adhered more closely to the Type-O diet (P<0.001).

Mean levels of cardiometabolic disease risk factors based on the tertiles of each diet score are shown from **Table 2** to **Table** **5** **.** All associations were adjusted for age, sex, ethnocultural group and energy intake. With increasing adherence to the Type-A diet, subjects, regardless of their ABO blood group, had lower BMI, blood pressure, waist circumference, serum total cholesterol, triglycerides, insulin, HOMA-IR, and HOMA-Beta (P<0.05). Adherence to the Type-AB diet was associated with lower blood pressure, serum total cholesterol, triglycerides, insulin, HOMA-IR, and HOMA-Beta (P<0.05). Adherence to the Type-O diet was associated with lower serum triglycerides (P<0.001). Although the overall association between the Type-B diet adherence and the level of HDL-cholesterol was significant (p = 0.04), no difference was observed between each tertile of the diet score.

**Table 2:** Cardiometabolic Risk Factors by the Tertiles of Type-A Diet Score^a^.

Cardiometabolic Risk Factors	T1	T2	T3	P-value
Body mass index (kg/m^2^)	23.7±0.2^b^	23.6±0.2	23.1±0.2	0.03^c^
Systolic blood pressure (mm Hg)	117.6±0.5	117.1±0.5	115.4±0.5	<0.001^d^
Diastolic blood pressure (mm Hg)	70.4±0.4	70.8±0.4	69.4±0.4	0.009^d^
Waist circumference (cm)	77.0±0.4	76.6±0.4	75.6±0.4	0.02^c^
Fasting glucose (mmol/L)	4.86±0.02	4.86±0.02	4.84±0.02	0.5
Fasting insulin (pmol/L)	52.7±1.8	53.3±1.8	46.3±1.8	0.002^d^
HOMA-IR	1.61±0.06	1.63±0.06	1.41±0.06	0.002^d^
HOMA-Beta	111.7±3.7	112.7±3.9	101.2±3.8	0.007^d^
Total cholesterol (mmol/L)	4.26±0.04	4.23±0.04	4.14±0.04	0.02^c^
HDL cholesterol (mmol/L)	1.46±0.02	1.47±0.02	1.45±0.02	0.35
LDL cholesterol (mmol/L)	2.34±0.03	2.31±0.03	2.28±0.03	0.34
Total:HDL cholesterol	3.06±0.04	3.05±0.04	3.03±0.04	0.64
Triglycerides (mmol/L)	1.00±0.02	0.99±0.02	0.92±0.02	0.005^c^
hs-CRP (mg/L)	1.53±0.13	1.44±0.14	1.07±0.13	0.06
Free fatty acids (μmol/L)	458.7±12.7	476.7±13.2	457.7±13.1	0.56

^a^ HDL, high density lipoprotein; LDL, low density lipoprotein; hs-CRP, high-sensitivity C-reactive protein; HOMA-IR, homeostasis model of insulin resistance; and HOMA-Beta, homeostasis model of beta-cell function. ANCOVA adjusted for age, sex, ethnicity and energy intake was used to examine associations between levels of cardiometabolic risk factors and tertiles of adherence scores in each ‘Blood-Type’ diet.

^b^ Mean ± SE (all such values).

^c^ T1>T3 after a Tukey-Kramer correction (P<0.05).

^d^ (T1, T2)>T3 after a Tukey-Kramer correction (P<0.05).

**Table 3:** Cardiometabolic Risk Factors by the Tertiles of Type-B Diet Score^a^.

Cardiometabolic Risk Factors	T1	T2	T3	P-value
Body mass index (kg/m^2^)	23.5±0.2^b^	23.4±0.2	23.5±0.2	0.77
Systolic blood pressure (mm Hg)	117.3±0.5	116.4±0.5	116.4±0.5	0.13
Diastolic blood pressure (mm Hg)	70.9±0.4	70.0±0.4	69.7±0.4	0.06
Waist circumference (cm)	76.8±0.4	76.1±0.4	76.4±0.4	0.54
Fasting glucose (mmol/L)	4.87±0.02	4.85±0.02	4.84±0.02	0.56
Fasting insulin (pmol/L)	51.3±1.8	51.4±1.8	50.1±1.8	0.35
HOMA-IR	1.57±0.06	1.56±0.06	1.53±0.06	0.35
HOMA-Beta	108.3±3.7	111.0±3.8	107.5±3.9	0.44
Total cholesterol (mmol/L)	4.25±0.04	4.2±0.04	4.17±0.04	0.21
HDL cholesterol (mmol/L)	1.47±0.02	1.47±0.02	1.43±0.02	0.04^c^
LDL cholesterol (mmol/L)	2.33±0.03	2.29±0.03	2.31±0.03	0.57
Total:HDL cholesterol	3.05±0.04	3.01±0.04	3.08±0.04	0.29
Triglycerides (mmol/L)	0.99±0.02	0.97±0.02	0.96±0.02	0.47
hs-CRP (mg/L)	1.39±0.13	1.24±0.13	1.45±0.14	0.5
Free fatty acids (μmol/L)	467.9±12.7	460.9±12.9	466.9±13.2	0.98

^a^ HDL, high density lipoprotein; LDL, low density lipoprotein; hs-CRP, high-sensitivity C-reactive protein; HOMA-IR, homeostasis model of insulin resistance; and HOMA-Beta, homeostasis model of beta-cell function. ANCOVA adjusted for age, sex, ethnicity and energy intake was used to examine associations between levels of cardiometabolic risk factors and tertiles of adherence scores in each ‘Blood-Type’ diet.

^b^ Mean ± SE (all such values).

^c^ Overall comparison is significantly different after a Tukey-Kramer correction (P<0.05).

**Table 4:** Cardiometabolic Risk Factors by the Tertiles of Type-AB Diet Scores^a^.

Cardiometabolic Risk Factors	T1	T2	T3	P-value
Body mass index (kg/m^2^)	23.7±0.2^b^	23.5±0.2	23.2±0.2	0.1
Systolic blood pressure (mm Hg)	117.3±0.5	117.1±0.5	115.5±0.5	0.006^c^
Diastolic blood pressure (mm Hg)	70.8±0.4	70.2±0.4	69.4±0.4	0.02^d^
Waist circumference (cm)	76.8±0.4	76.7±0.4	75.7±0.4	0.08
Fasting glucose (mmol/L)	4.88±0.02	4.83±0.02	4.84±0.02	0.13
Fasting insulin (pmol/L)	56.1±1.7	49.8±1.8	45.6±1.9	<0.001^e^
HOMA-IR	1.72±0.06	1.52±0.06	1.39±0.06	<0.001^e^
HOMA-Beta	119.0±3.7	107.0±3.8	98.0±3.9	<0.001^c^
Total cholesterol (mmol/L)	4.27±0.04	4.19±0.04	4.16±0.04	0.03^d^
HDL cholesterol (mmol/L)	1.47±0.02	1.45±0.02	1.46±0.02	0.72
LDL cholesterol (mmol/L)	2.35±0.03	2.30±0.03	2.28±0.03	0.19
Total:HDL cholesterol	3.07±0.04	3.05±0.04	3.02±0.04	0.31
Triglycerides (mmol/L)	1.01±0.02	0.96±0.02	0.93±0.02	0.004^f^
hs-CRP (mg/L)	1.45±0.13	1.48±0.13	1.11±0.14	0.08
Free fatty acids (μmol/L)	464.9±12.5	470.4±12.9	459.9±13.5	0.76

^a^ HDL, high density lipoprotein; LDL, low density lipoprotein; hs-CRP, high-sensitivity C-reactive protein; HOMA-IR, homeostasis model of insulin resistance; and HOMA-Beta, homeostasis model of beta-cell function. ANCOVA adjusted for age, sex, ethnicity and energy intake was used to examine associations between levels of cardiometabolic risk factors and tertiles of adherence scores in each ‘Blood-Type’ diet.

^b^ Mean ± SE (all such values).

^c^ (T1, T2)>T3 after a Tukey-Kramer correction (P<0.05).

^d^ T1>T3 after a Tukey-Kramer correction (P<0.05).

^e^ T1>T2>T3 after a Tukey-Kramer correction (P<0.05).

^f^ T1>(T2, T3) after a Tukey-Kramer correction (P<0.05).

**Table 5:** Cardiometabolic Risk Factors by the Tertiles of Type-O Diet Scores^a^.

Cardiometabolic Risk Factors	T1	T2	T3	P-value
Body mass index (kg/m^2^)	23.6±0.2^b^	23.5±0.2	23.4±0.2	0.79
Systolic blood pressure (mm Hg)	116.8±0.5	116.8±0.5	116.6±0.5	0.9
Diastolic blood pressure (mm Hg)	70.3±0.4	69.9±0.4	70.4±0.4	0.67
Waist circumference (cm)	77.0±0.4	76.5±0.4	75.8±0.4	0.12
Fasting glucose (mmol/L)	4.84±0.02	4.85±0.02	4.87±0.02	0.6
Fasting insulin (pmol/L)	51.1±1.9	50.9±1.8	50.9±1.8	0.93
HOMA-IR	1.56±0.06	1.56±0.06	1.56±0.06	0.96
HOMA-Beta	110.0±4.0	110.2±3.8	106.7±3.9	0.7
Total cholesterol (mmol/L)	4.28±0.04	4.15±0.04	4.2±0.04	0.054
HDL cholesterol (mmol/L)	1.46±0.02	1.46±0.02	1.46±0.02	0.83
LDL cholesterol (mmol/L)	2.36±0.03	2.26±0.03	2.33±0.03	0.06
Total:HDL cholesterol	3.12±0.04	3.00±0.04	3.03±0.04	0.09
Triglycerides (mmol/L)	1.04±0.03	0.96±0.02	0.91±0.02	<0.001^c^
hs-CRP (mg/L)	1.49±0.14	1.41±0.13	1.2±0.14	0.14
Free fatty acids (μmol/L)	464.6±13.7	452.1±12.8	479.0±13.2	0.39

^a^ HDL, high density lipoprotein; LDL, low density lipoprotein; hs-CRP, high-sensitivity C-reactive protein; HOMA-IR, homeostasis model of insulin resistance; and HOMA-Beta, homeostasis model of beta-cell function. ANCOVA adjusted for age, sex, ethnicity and energy intake was used to examine associations between levels of cardiometabolic risk factors and tertiles of adherence scores in each ‘Blood-Type’ diet.

^b^ Mean ± SE (all such values).

^c^ (T1>(T2, T3) after a Tukey-Kramer correction (P<0.05).

**Table 6** **,** **7** **,** **8** **and** **9** show the associations between diet scores and cardiometabolic disease risk factors according to the ABO blood group. Different ABO blood groups were equally distributed across the tertiles of each diet score. No significant interactions were observed between diet score and blood group for most of the risk factors, except for fasting glucose (P = 0.02), insulin (P = 0.02), and HOMA-IR (p = 0.01) in the Type-A diet **(** **Table 6** **)**, and fasting glucose (P = 0.02) in the Type-AB diet **(** **Table 8** **)**. When comparing the levels of fasting insulin and HOMA-IR between group A individuals and the other blood groups, a significant difference was observed in the second tertile, but not in the lowest or highest tertile of the Type-A diet score. No difference in fasting glucose was observed between the two groups in any tertile of the Type-A diet score. For fasting glucose in the Type-AB diet, no difference was observed between individuals with blood group AB and those with other blood groups in any tertile.

**Table 6:** Cardiometabolic Disease Risk Factors by Matching Type-A Diet Scores and ABO Genotype^a^.

ABO Genotype		A			B/AB/O		P-value for ABO * Diet interaction
Type-A Diet Score Tertile	T1	T2	T3	T1	T2	T3	
Subjects [n (% of total)]	177 (33)	182 (33)	185 (34)	307 (34)	295 (32)	309 (34)	
Body mass index (kg/m^2^)	23.1±0.3^b^	23.6±0.3	23.0±0.3	24.0±0.2	23.5±0.2	23.1±0.2	0.15
Systolic blood pressure (mm Hg)	117.1±0.7	117.4±0.7	115.8±0.7	117.9±0.6	116.9±0.6	115.2±0.6	0.48
Diastolic blood pressure (mm Hg)	69.6±0.6	70.8±0.6	68.6±0.6	70.8±0.5	70.7±0.5	69.8±0.5	0.39
Waist circumference (cm)	76.2±0.6	76.7±0.6	75.2±0.6	77.5±0.5	76.4±0.5	75.8±0.5	0.32
Glucose (mmol/L)	4.82±0.03	4.91±0.03	4.83±0.03	4.88±0.02	4.84±0.02	4.84±0.02	0.02^c^
Insulin (pmol/L)	51.9±2.8	60.5±2.7	47.7±2.8	54.4±2.1	48.6±2.2	45.6±2.1	0.02^d^
HOMA-IR	1.57±0.09	1.87±0.09	1.45±0.09	1.68±0.07	1.48±0.07	1.39±0.07	0.01^d^
HOMA-Beta	114.6±5.9	123.4±5.8	105.2±5.9	112.9±4.5	105.8±4.6	99.1±4.5	0.26
Total cholesterol (mmol/L)	4.37±0.06	4.34±0.06	4.15±0.06	4.21±0.05	4.16±0.05	4.14±0.05	0.09
HDL cholesterol (mmol/L)	1.51±0.03	1.47±0.03	1.46±0.03	1.44±0.02	1.46±0.02	1.44±0.02	0.31
LDL cholesterol (mmol/L)	2.42±0.05	2.4±0.05	2.29±0.05	2.3±0.04	2.26±0.04	2.28±0.04	0.16
Total:HDL cholesterol	3.01±0.06	3.10±0.06	3.00±0.06	3.09±0.04	3.02±0.05	3.04±0.04	0.25
Triglycerides (mmol/L)	0.96±0.04	1.01±0.04	0.9±0.04	1.02±0.03	0.97±0.03	0.93±0.03	0.15
hs-CRP (mg/L)	1.48±0.21	1.56±0.2	1.02±0.2	1.62±0.16	1.34±0.16	1.09±0.16	0.25
Free fatty acids (μmol/L)	449.1±19.9	481.6±19.8	471.1±19.9	474.3±15.2	470.2±15.8	448.5±15.4	0.24

^a^ HDL, high density lipoprotein; LDL, low density lipoprotein; hs-CRP, high-sensitivity C-reactive protein; HOMA-IR, homeostasis model of insulin resistance; and HOMA-Beta, homeostasis model of beta-cell function. ANCOVA adjusted for age, sex, ethnicity and energy intake was used to examine the interaction effect between the ABO blood group and diet adherence on levels of cardiometabolic risk factors. The Tukey-Kramer procedure was used to adjust for multiple comparisons between groups within each ANCOVA.

^b^ Mean ± SE (all such values).

^c^ Overall interaction is significant after a Tukey-Kramer correction (P<0.05).

^d^ (T2 in Blood Group A) > (T2 in Group B/AB/O) after a Tukey-Kramer correction (P<0.05).

**Table 7:** Cardiometabolic Risk Factors by Matching Type-B Diet Scores and ABO Genotype^a^.

ABO Genotype	B			A/AB/O			P-value for ABO * Diet interaction
Type-B Diet Score Tertile	T1	T2	T3	T1	T2	T3	
Subjects [n (% of total)]	87 (31)	103 (38)	87 (31)	395 (34)	383 (32)	400 (34)	
Body mass index (kg/m^2^)	23.9±0.4^b^	22.7±0.4	23.8±0.4	23.5±0.2	23.5±0.2	23.4±0.2	0.06
Systolic blood pressure (mm Hg)	116.5±1.0	115.4±0.9	116.6±1.0	117.5±0.5	116.6±0.5	116.3±0.5	0.51
Diastolic blood pressure (mm Hg)	69.8±0.9	69.8±0.8	70.4±0.9	71.1±0.4	70.0±0.5	69.5±0.5	0.22
Waist circumference (cm)	76.7±0.9	75.0±0.8	76.7±0.9	76.8±0.4	76.4±0.5	76.4±0.5	0.34
Glucose (mmol/L)	4.85±0.04	4.85±0.04	4.82±0.04	4.87±0.02	4.84±0.02	4.85±0.02	0.81
Insulin (pmol/L)	54.1±3.8	52.2±3.5	57.9±3.8	50.7±1.9	51.1±2.0	48.4±2.0	0.9
HOMA-IR	1.67±0.12	1.59±0.11	1.78±0.12	1.55±0.06	1.55±0.06	1.48±0.06	0.93
HOMA-Beta	114.3±8.1	112.4±7.4	122.1±8.1	106.8±4.1	110.5±4.2	104.3±4.2	0.78
Total cholesterol (mmol/L)	4.21±0.08	4.19±0.08	4.13±0.08	4.26±0.04	4.21±0.04	4.19±0.04	0.96
HDL cholesterol (mmol/L)	1.43±0.04	1.51±0.04	1.42±0.04	1.48±0.02	1.46±0.02	1.43±0.02	0.09
LDL cholesterol (mmol/L)	2.32±0.07	2.26±0.06	2.26±0.07	2.33±0.04	2.30±0.04	2.33±0.04	0.88
Total:HDL cholesterol	3.10±0.08	2.92±0.07	3.11±0.08	3.04±0.04	3.04±0.04	3.08±0.04	0.2
Triglycerides (mmol/L)	1.01±0.05	0.92±0.05	0.99±0.05	0.98±0.03	0.98±0.03	0.95±0.03	0.53
hs-CRP (mg/L)	1.39±0.28	1.02±0.26	1.36±0.28	1.40±0.14	1.30±0.15	1.48±0.15	0.61
Free fatty acids (μmol/L)	490.3±27.4	423.8±25.1	471.6±27.6	463.6±13.8	471.5±14.3	466.6±14.2	0.28

^a^ HDL, high density lipoprotein; LDL, low density lipoprotein; hs-CRP, high-sensitivity C-reactive protein; HOMA-IR, homeostasis model of insulin resistance; and HOMA-Beta, homeostasis model of beta-cell function. ANCOVA adjusted for age, sex, ethnicity and energy intake was used to examine the interaction effect between the ABO blood group and diet adherence on levels of cardiometabolic risk factors. The Tukey-Kramer procedure was used to adjust for multiple comparisons between groups within each ANCOVA.

^b^ Mean ± SE (all such values).

**Table 8:** Cardiometabolic Risk Factors by Matching Type-AB Diet Scores and ABO Genotype^a^.

ABO Genotype	AB			A/B/O			P-value for ABO * Diet interaction
Type-AB Diet Score Tertile	T1	T2	T3	T1	T2	T3	
Subjects [n (% of total)]	26 (29)	35 (38)	30 (33)	464 (34)	441 (32)	459 (34)	
Body mass index (kg/m^2^)	24.3±0.7^b^	25.1±0.6	22.7±0.6	23.6±0.2	23.4±0.2	23.2±0.2	0.07
Systolic blood pressure (mm Hg)	116.4±1.8	118.9±1.6	114.9±1.7	117.3±0.5	116.9±0.5	115.6±0.5	0.39
Diastolic blood pressure (mm Hg)	71.4±1.6	70.6±1.4	69.4±1.5	70.8±0.4	70.2±0.4	69.4±0.4	0.96
Waist circumference (cm)	77.5±1.6	78.9±1.4	74.2±1.5	76.8±0.4	76.5±0.4	75.8±0.4	0.14
Glucose (mmol/L)	4.74±0.07	4.84±0.06	4.95±0.06	4.89±0.02	4.83±0.02	4.84±0.02	0.02^c^
Insulin (pmol/L)	56.6±6.8	48.4±5.9	41.7±6.3	56.0±1.8	50.0±1.8	45.9±1.9	0.81
HOMA-IR	1.67±0.22	1.48±0.19	1.29±0.20	1.72±0.06	1.52±0.06	1.4±0.06	0.94
HOMA-Beta	133.6±14.3	101.8±12.4	82.7±13.4	118.2±3.8	107.4±3.9	99.0±4.1	0.15
Total cholesterol (mmol/L)	4.29±0.15	4.14±0.13	4.10±0.14	4.27±0.04	4.19±0.04	4.16±0.04	0.91
HDL cholesterol (mmol/L)	1.45±0.07	1.42±0.06	1.49±0.06	1.47±0.02	1.45±0.02	1.46±0.02	0.8
LDL cholesterol (mmol/L)	2.35±0.12	2.32±0.11	2.22±0.12	2.35±0.03	2.30±0.03	2.28±0.04	0.85
Total:HDL cholesterol	3.17±0.14	3.00±0.12	2.94±0.13	3.07±0.04	3.05±0.04	3.02±0.04	0.73
Triglycerides (mmol/L)	1.09±0.09	0.87±0.08	0.86±0.08	1.01±0.02	0.96±0.02	0.94±0.03	0.26
hs-CRP (mg/L)	1.68±0.5	2.48±0.44	1.03±0.47	1.44±0.13	1.39±0.14	1.11±0.14	0.21
Free fatty acids (μmol/L)	456.3±48.9	529.4±42.4	400.1±45.7	465.7±12.8	466.1±13.3	464.3±13.8	0.11

^a^ HDL, high density lipoprotein; LDL, low density lipoprotein; hs-CRP, high-sensitivity C-reactive protein; HOMA-IR, homeostasis model of insulin resistance; and HOMA-Beta, homeostasis model of beta-cell function. ANCOVA adjusted for age, sex, ethnicity and energy intake was used to examine the interaction effect between the ABO blood group and diet adherence on levels of cardiometabolic risk factors. The Tukey-Kramer procedure was used to adjust for multiple comparisons between groups within each ANCOVA.

^b^ Mean ± SE (all such values).

^c^ Overall interaction is significant after a Tukey-Kramer correction (P<0.05).

**Table 9:** Cardiometabolic Risk Factors by Matching Type-O Diet Scores and ABO Genotype^a^.

ABO Genotype	O			A/B/AB			P-value for ABO * Diet interaction
Type-O Diet Score Tertile	T1	T2	T3	T1	T2	T3	
Subjects [n (% of total)]	182 (34)	182 (34)	179 (32)	300 (33)	319 (35)	293 (32)	
Body mass index (kg/m^2^)	23.8±0.3^b^	23.6±0.3	23.3±0.3	23.4±0.2	23.4±0.2	23.4±0.2	0.83
Systolic blood pressure (mm Hg)	117.0±0.7	117.2±0.7	116.5±0.7	116.6±0.6	116.6±0.6	116.6±0.6	0.75
Diastolic blood pressure (mm Hg)	71.5±0.6	70.4±0.6	70.2±0.6	69.6±0.5	69.7±0.5	70.4±0.5	0.14
Waist circumference (cm)	77.4±0.6	76.9±0.6	76.3±0.6	76.8±0.5	76.2±0.5	75.5±0.5	0.93
Glucose (mmol/L)	4.86±0.03	4.83±0.03	4.88±0.03	4.83±0.02	4.86±0.02	4.86±0.02	0.39
Insulin (pmol/L)	48.0±2.8	46.3±2.7	47.5±2.7	53.1±2.3	53.9±2.2	53.3±2.2	0.79
HOMA-IR	1.46±0.09	1.41±0.09	1.45±0.09	1.62±0.07	1.65±0.07	1.64±0.07	0.72
HOMA-Beta	103.5±5.8	99.2±5.7	100.8±5.8	114.3±4.8	117.1±4.6	111.2±4.7	0.98
Total cholesterol (mmol/L)	4.26±0.06	4.10±0.06	4.13±0.06	4.30±0.05	4.18±0.05	4.25±0.05	0.76
HDL cholesterol (mmol/L)	1.45±0.03	1.45±0.03	1.42±0.03	1.46±0.02	1.46±0.02	1.49±0.02	0.43
LDL cholesterol (mmol/L)	2.33±0.05	2.20±0.05	2.30±0.05	2.37±0.04	2.29±0.04	2.34±0.04	0.85
Total:HDL cholesterol	3.16±0.06	2.99±0.06	3.06±0.06	3.10±0.05	3.00±0.05	3.01±0.05	0.68
Triglycerides (mmol/L)	1.07±0.04	0.98±0.04	0.91±0.04	1.03±0.03	0.95±0.03	0.91±0.03	0.65
hs-CRP (mg/L)	1.48±0.20	1.25±0.20	1.34±0.20	1.50±0.17	1.50±0.16	1.12±0.16	0.81
Free fatty acids (μmol/L)	470.7±19.9	446.2±19.2	480.8±19.7	460.8±16.4	455.5±15.5	477.9±16.0	0.89

^a^ HDL, high density lipoprotein; LDL, low density lipoprotein; hs-CRP, high-sensitivity C-reactive protein; HOMA-IR, homeostasis model of insulin resistance; and HOMA-Beta, homeostasis model of beta-cell function. ANCOVA adjusted for age, sex, ethnicity and energy intake was used to examine the interaction effect between the ABO blood group and diet adherence on levels of cardiometabolic risk factors. The Tukey-Kramer procedure was used to adjust for multiple comparisons between groups within each ANCOVA.

^b^ Mean ± SE (all such values).

## Discussion

Our findings show that adherence to certain ‘Blood-Type’ diets is associated with a favorable profile for certain cardiometabolic risk factors in young adults, but these associations were not related to an individual's ABO blood group. To our knowledge, this is the first study to examine the association between the ‘Blood-Type’ diets and biomarkers of cardiometabolic health, and the findings do not support the ‘Blood-Type’ diet hypothesis.

The association between the Type-A diet adherence and favorable cardiometabolic risk profile is not surprising considering this diet's emphasis on high consumption of fruits and vegetables, and low consumption of meat products, which is similar to a dietary pattern that has been recommended by various health agencies because of its association with a lower risk of cardiovascular diseases [19], [20], [21], [22], [23]. Adherence to the Type-AB diet was also associated with favorable levels of several risk factors, despite its recommendation for certain dairy and meat products. Such benefits may be attributed to the list of certain food items considered healthy, which are recommended. For example, individuals with blood group AB are advised to avoid butter and to consume eggs and fish as their main animal-protein source. This is in contrast to the Type-B diet, which has fewer restrictions on many animal products as shown in the **Appendix S1**. These differences between the two diets may partially explain why a favorable cardiometabolic profile was associated with adherence to the Type-AB diet, but not for the Type-B diet. The Type-O diet is similar to low-carbohydrate diets [24], which may explain why adherence to this type of diet was associated with lower serum triglycerides (TG), as previously observed for other low-carbohydrate diets [25], [26]. The reduction in TG may be caused by decreased TG production in the liver and/or increased cellular uptake of TG in response to low carbohydrate intake [27]. By investigating the ‘Blood-Type’ diets in a population with different *ABO* genotypes, we found that adhering to the Type-A, Type-AB, or Type-O diets was associated with favorable effects on levels of certain biomarkers of cardiometabolic disease risk.

In order to examine whether individuals would benefit more from following their own ‘Blood-Type’ diet, the levels of cardiometabolic disease risk factors were compared between individuals with the matched blood group and the unmatched blood group while sharing similar diet adherence. However, no significant interaction effects were observed between diet adherence and blood group for most of the risk factors, suggesting that effects of following ‘Blood-Type’ diets is independent of an individual's blood group. Although there were significant interaction effects for fasting glucose, insulin and HOMA-IR for the Type-A diet, and fasting glucose for the Type-AB diet, those interactions may be due to chance, since we did not apply the most conservative Bonferroni post-hoc test to correct for multiple comparisons. Even if the interaction effects were not due to chance, those findings would not support the claim that matching the ‘Blood-Type’ diet with the corresponding blood group results in more favorable effects. In the case of the Type-A diet, the significant interaction effects were mainly driven by higher levels of insulin and HOMA-IR in the second tertile for those with blood group A. Moving from low adherence to high adherence, group A individuals did not demonstrate more favorable changes in these biomarkers. As for fasting glucose levels with the Type-AB diet, subjects with blood group AB had slightly higher glucose concentrations as they adhered to the diet more closely, while the other blood groups showed no differences. These findings, therefore, demonstrate that matching the diet with the corresponding blood group was not associated with any additional benefits and may even be associated with some adverse effects. For those in the unmatched blood group, we also tested whether each ‘Blood-Type’ diet was associated with any of the outcomes by matching to each of the other blood groups (data not shown); however, no significant interactions were observed. Therefore, the associations observed with the ‘Blood-Type’ diets were unrelated to any individual blood group.

Several previous studies have questioned the validity of the ‘Blood-Type’ diets. Based on phylogenetic analysis of human ABO alleles, blood group A has been suggested to be the ancestral human blood group [28], [29], rather than group O as postulated by D'Adamo [1]. As for the claim that certain food items contain lectins incompatible with an individual's ABO blood group, studies to date suggest no ABO-specific agglutination [30]. The absence of scientific evidence was further supported by a recent systematic review [14], which found no study that directly investigated the effects of the ‘Blood-Type’ diet.

The present study has some limitations. The use of FFQs for dietary assessment could result in some measurement error and cannot give a precise estimate of the absolute intake of food items. However, a FFQ is considered a valid instrument for providing relative estimates of food intake in large populations [31]. Although we adjusted for age, sex, ethnocultural group and energy intake and tested physical activity and smoking as potential covariates, the observed associations between ‘Blood-Type’ diet scores and cardiometabolic disease risk factors could be due to residual confounding. However, residual confounding is not likely to explain why there would be no differential association among *ABO* genotypes. The study population consisted of an unequal distribution of different ethnocultural groups, which have been shown to have a different prevalence of ABO blood groups [32] and might have different dietary patterns [16]. However, the associations between diet adherence and levels of biomarkers were still evident after adjusting for ethnocultural group. Previous studies using diet scores have quantified relative adherence by deriving the score proportionally based on the recommended amount of consumption [33]. However, this approach would not be appropriate for quantifying the adherence to the ‘Blood-Type’ diet because its recommendations do not specify any actual amount of consumption. By assigning points based on quantity of consumption for each food item, our scoring system is continuously scaled and normally distributed. Since the scoring system in the present study only assessed relative adherence to each of the four ‘Blood-Type’ diets, we could not determine the absolute number of people who strictly followed any of the diets. However, the observed results showed that even relatively high adherence to Type-A, Type-AB and Type-O diets were associated with favorable levels of cardiometabolic disease risk factors, albeit in an ABO-independent manner. These associations were consistent with previous studies examining similar dietary patterns and cardiometabolic risk factors [23], [24], [34].

In summary, the present study is the first to test the validity of the ‘Blood-Type’ diet and we showed that adherence to certain diets is associated with some favorable cardiometabolic disease risk profiles. This may explain anecdotal evidence supporting these diets, which are generally prudent diets that reflect healthy eating habits. However, the findings showed that the observed associations were independent of ABO blood group and, therefore, the findings do not support the ‘Blood-Type’ diet hypothesis.

## Supporting Information

Table S1 **The ‘Blood-Type’ Diet Characteristics.**
(DOCX)

Appendix S1 **The food list was retrieved from the FFQ database of Toronto Nutrigenomics and Health Study.** The “+” signs indicate the foods that are recommended for the blood group. The “-” signs indicate the food to avoid for the blood group. The “/” signs indicate the food that are neutral.
(PDF)
