# Supplementary material for: ABO Genotype, ‘Blood-Type’ Diet and Cardiometabolic Risk Factors
Source: PLoS One. 2014 Jan 15;9(1):e84749. doi: 10.1371/journal.pone.0084749 (PMC3893150; doi:10.1371/journal.pone.0084749)
Supplement: Table S1 — The ‘Blood-Type’ Diet Characteristics. (DOCX) [file pone.0084749.s001.docx]

| **Table S1: The ‘Blood-Type’ Diet Characteristics^a^** | | | | |  | |  | |  | |  | |  |
| --- | --- | --- | --- | --- | --- | --- | --- | --- | --- | --- | --- | --- | --- |
|  | **Type-A Diet Score** | | | |  | | **Type-B Diet Score** | | | | | |  |
| **Diet Characteristics** | **T1** | **T2** | **T3** | **P-value** | | **T1** | | **T2** | | **T3** | | **P-value** | |
| Total energy intake (Kcal) | 2163 ± 29^b^ | 1857 ± 29 | 1880 ± 29 | <0.001^c^ | | 1934 ± 30 | | 1890 ± 29 | | 2076 ± 29 | | <0.001^d^ | |
| Fruit and vegetables, servings/day | 6.1 ± 0.2 | 6.3 ± 0.2 | 8.3 ± 0.2 | <0.001^d^ | | 5.3 ± 0.2 | | 6.4 ± 0.2 | | 9.1 ± 0.2 | | <0.001^e^ | |
| Cereal, servings/day | 3.8 ± 0.1 | 3.3 ± 0.1 | 3.5 ± 0.1 | <0.001^c^ | | 3.5 ± 0.1 | | 3.4 ± 0.1 | | 3.5 ± 0.1 | | 0.56 | |
| Meat, servings/day | 2.3 ± 0.1 | 1.7 ± 0.1 | 1.4 ± 0.1 | <0.001^c^ | | 2.1 ± 0.1 | | 1.7 ± 0.1 | | 1.7 ± 0.1 | | <0.001^c^ | |
| Dairy, servings/day | 2.4 ± 0.1 | 1.9 ± 0.1 | 1.8 ± 0.1 | <0.001^c^ | | 1.8 ± 0.1 | | 2.0 ± 0.1 | | 2.3 ± 0.1 | | <0.001^c^ | |
| Energy from carbohydrate (%) | 50.1 ± 0.4 | 52.5 ± 0.4 | 55.8 ± 0.4 | <0.001^e^ | | 50.4 ± 0.4 | | 53.1 ± 0.4 | | 54.9 ± 0.4 | | <0.001^e^ | |
| Energy from total fat (%) | 31.3 ± 0.3 | 30.1 ± 0.3 | 28.4 ± 0.3 | <0.001^e^ | | 31.0 ± 0.3 | | 29.6 ± 0.3 | | 29.0 ± 0.3 | | <0.001^c^ | |
| Energy from animal fat (%) | 17.6 ± 0.2 | 15.0 ± 0.2 | 11.4 ± 0.2 | <0.001^e^ | | 15.6 ± 0.3 | | 14.6 ± 0.3 | | 13.6 ± 0.2 | | <0.001^e^ | |
| Energy from vegetable fat (%) | 13.8 ± 0.3 | 15.1 ± 0.3 | 17.0 ± 0.3 | <0.001^e^ | | 15.4 ± 0.3 | | 15.1 ± 0.3 | | 15.5 ± 0.3 | | 0.57 | |
| Fiber (g) | 21.0 ± 0.5 | 21.3 ± 0.5 | 28.2 ± 0.5 | <0.001^d^ | | 19.7 ± 0.5 | | 22.3 ± 0.5 | | 28.6 ± 0.5 | | <0.001^e^ | |

^a^ Differences among tertiles of each diet score were assessed by analysis of variance.

^b^ Mean ± SE (all such values).

^c^ T1 > (T2, T3) after a Tukey-Kramer correction (P<0.05).

^d^ T3 > (T1, T2) after a Tukey-Kramer correction (P<0.05).

^e^ T3 > T2 > T1 after a Tukey-Kramer correction (P<0.05).

^f^ T3 > T2 after a Tukey-Kramer correction (P<0.05).

^g^ T1 > T2 > T3 after a Tukey-Kramer correction (P<0.05).

^h^ (T3, T2) > T1 after a Tukey-Kramer correction (P<0.05).

| **Table S1: The ‘Blood-Type’ Diet Characteristics (continued)** | | | | |  |  |  |
| --- | --- | --- | --- | --- | --- | --- | --- |
| **Type-AB Diet Score** | | |  | **Type-O Diet Score** | | |  |
| **T1** | **T2** | **T3** | **P-value** | **T1** | **T2** | **T3** | **P-value** |
| 1964 ± 29 | 1901 ± 30 | 2033 ± 29 | 0.007^f^ | 2247 ± 28 | 1868 ± 28 | 1784 ± 29 | <0.001^c^ |
| 5.2 ± 0.2 | 6.5 ± 0.2 | 9.1 ± 0.2 | <0.001^e^ | 7.5 ± 0.2 | 6.3 ± 0.2 | 6.9 ± 0.2 | <0.001^c^ |
| 3.5 ± 0.1 | 3.3 ± 0.1 | 3.7 ± 0.1 | 0.018^f^ | 4.3 ± 0.1 | 3.3 ± 0.1 | 2.8 ± 0.1 | <0.001^g^ |
| 2.2 ± 0.1 | 1.8 ± 0.1 | 1.5 ± 0.1 | <0.001^g^ | 1.6 ± 0.1 | 1.7 ± 0.1 | 2.1 ± 0.1 | <0.001^d^ |
| 1.9 ± 0.1 | 2.0 ± 0.1 | 2.2 ± 0.1 | 0.002^d^ | 2.7 ± 0.1 | 2.0 ± 0.1 | 1.5 ± 0.1 | <0.001^g^ |
| 50.6 ± 0.4 | 52.6 ± 0.4 | 55.3 ± 0.4 | <0.001^e^ | 54.1 ± 0.4 | 52.8 ± 0.4 | 51.5 ± 0.4 | <0.001^g^ |
| 30.9 ± 0.3 | 29.7 ± 0.3 | 29.1 ± 0.3 | <0.001^c^ | 29.1 ± 0.3 | 30.1 ± 0.3 | 30.5 ± 0.3 | 0.003^h^ |
| 17.4 ± 0.2 | 14.9 ± 0.2 | 11.4 ± 0.2 | <0.001^g^ | 13.6 ± 0.3 | 14.7 ± 0.2 | 15.4 ± 0.2 | <0.001^h^ |
| 13.4 ± 0.3 | 14.8 ± 0.3 | 17.7 ± 0.3 | <0.001^e^ | 15.5 ± 0.3 | 15.4 ± 0.3 | 15.0 ± 0.3 | 0.52 |
| 17.8 ± 0.5 | 22.3 ± 0.5 | 30.5 ± 0.5 | <0.001^e^ | 27.1 ± 0.5 | 21.9 ± 0.5 | 21.7 ± 0.5 | <0.001^c^ |
