# Supplementary material for: ABO Genotype, ‘Blood-Type’ Diet and Cardiometabolic Risk Factors
Source: PLoS One. 2014 Jan 15;9(1):e84749. doi: 10.1371/journal.pone.0084749 (PMC3893150; doi:10.1371/journal.pone.0084749)
Supplement: Appendix S1 — The food list was retrieved from the FFQ database of Toronto Nutrigenomics and Health Study. The “+” signs indicate the foods that are recommended for the blood group. The “-” signs indicate the food to avoid for the blood group. The “/” signs indicate the food that are neutral. (PDF) [file pone.0084749.s002.pdf]

Appendix S1

	**ABO Blood Group**			
**Food List**	A	B	AB	O
Skim milk	-	+	/	-
Milk 1-2%	-	+	/	-
Whole milk	-	+	-	-
Soy milk	+	-	/	/
Flavoured yogurt	/	+	+	-
Plain yogurt	/	+	+	-
Cottage cheese	-	+	+	-
Cream cheese	-	/	/	-
Other cheese	/	+	+	/
Butter	-	/	-	/
Grapes	/	+	+	/
Prunes	+	/	/	+
Bananas	-	+	-	+
Cantaloupe	/	/	/	-
Honeydew	-	/	/	-
Watermelon	/	+	+	/
Avocado	/	-	-	-
Apple	/	/	/	/
Apple juice	/	/	/	/
Orange	-	/	-	-
Orange juice	-	/	-	-
Grapefruit	+	/	+	/
Grapefruit juice	+	/	/	/
Other fruit juices	/	+	+	/
Strawberries	/	/	/	/
Blueberries	+	/	/	+
Peaches	/	/	/	/
Tropical fruits	-	+	+	/
Dried apricot	+	/	/	/
Raisins	/	/	/	/
Dried dates	/	/	/	/
Cranberries	+	+	+	/
Tomatoes	-	-	/	/
Tomato juice	-	-	/	/
Tomato sauce	-	-	/	/
Tofu	+	-	+	/
String beans	+	/	/	/
Broccoli	+	+	+	+
Cabbage	-	+	/	/
Cauliflower	/	+	+	-
Brussels Sprouts	/	+	/	/
Raw carrots	+	+	/	/
Cooked carrots	+	+	/	/
Corn	/	-	-	-
Peas	/	/	/	/
Mixed vegetables	+	+	+	+
Beans	+	-	/	-
Squash	/	/	/	/
Zucchini	/	/	/	/
Eggplant	-	+	+	/
Yams	-	+	+	/
Cooked spinach	+	/	/	+
Raw spinach	+	/	/	+
Kale	+	+	+	+
Iceberg	/	/	/	/
Romaine lettuce	+	/	/	+
Celery	+	/	+	/
Green peppers	-	+	-	/
Red peppers	-	+	-	+
Onions (Garnish)	+	/	/	+
Onions	+	/	/	+
Okra	+	/	/	+
Cucumber	/	/	+	-
Olives	-	-	-	-
Beets	/	+	+	/
Asparagus	/	/	/	/
Egg whites	/	/	/	/
Eggs	/	/	/	/
Bacon	-	-	-	-
Chicken sandwich	/	-	-	/
Chicken/Turkey with skin	/	-	-	/
Chicken/Turkey without skin	/	-	-	/
Hotdogs	-	/	-	+
Chicken/Turkey hotdogs	/	-	-	/
Processed meat sandwich	-	/	-	+
Processed meat	-	/	-	+
Lean hamburger	-	/	-	+
Hamburger	-	/	-	+
Sandwich	-	/	-	+
Pork	-	-	-	-
Beef	-	/	-	+
Liver	-	/	/	+
Chicken liver	/	-	-	/
Other organ meats	-	/	-	+
Canned tuna	/	/	+	/
Fish cakes	+	+	+	+
Shrimp	-	-	-	/
Dark meat fish	+	+	+	/
Other fish	+	+	+	+
Cold cereal	/	-	/	-
Cooked oatmeal	+	+	+	/
Cooked oat bran	+	+	+	/
Cooked breakfast cereal	/	-	-	-
White bread	/	/	/	-
Muffins	-	/	/	-
Heavy bread	+	-	+	/
Dark bread	/	-	/	/
Bagel	/	/	/	-
Biscuits	/	/	/	-
Brown rice	/	/	+	/
White rice	/	/	+	/
Mac & cheese	/	/	/	-
Pasta	/	/	/	-
Whole grain pasta	-	-	/	-
Barley	/	/	/	-
Bulgur	/	-	/	-
Other grains	/	/	/	-
Pancakes	/	/	/	-
French fries	-	/	/	-
Potatoes (scalloped)	-	/	/	-
Potatoes (baked)	-	/	/	-
Potato chips	-	/	/	-
Corn chips	/	-	-	-
Crackers	/	/	/	-
Pizza	/	/	/	-
Tortillas	/	/	/	-
